# Calorie restriction regime enhances physical performance of trained athletes

**DOI:** 10.1186/s12970-018-0214-2

**Published:** 2018-03-09

**Authors:** Victoria Pons, Joan Riera, Xavier Capó, Miquel Martorell, Antoni Sureda, Josep A. Tur, Franchek Drobnic, Antoni Pons

**Affiliations:** 1 Sport Nutrition and Physiology Dept, Olympic Training Center, CAR – GIRSANE, Sant Cugat del Vallés, Spain; 2 Research Group on Community Nutrition and Oxidative Stress, Science Laboratory of Physical Activity, Department of Fundamental Biology and Health Sciences, University of Balearic Islands, 07122 Palma de Mallorca, Spain; 3 CIBER: CB12/03/30038 Fisiopatología de la Obesidad la Nutrición, CIBEROBN, Instituto de Salud Carlos III (ISCIII), University of Balearic Islands, 07122 Palma de Mallorca, Spain; 4 Departamento de Nutrición y Dietética, Facultad de Farmacia, Universidad de Concepción, 4070386 Concepción, Chile

**Keywords:** Caloric restriction, Physical performance, Fatty acids, Body composition

## Abstract

**Background:**

Caloric restriction induces mitochondrial biogenesis and improves physical fitness in rodents. We aimed to provide evidence of how caloric restriction affects the body composition and physical performance of trained athletes and to evaluate the possible impact of an every-other-day feeding diet on nutritional deficiencies of micronutrients and essential fatty acids.

**Methods:**

The study was performed with 12 healthy male athletes by carrying out a 33% caloric restriction with respect to their usual diet. Athletes performed a maximal exercise stress test both before and after the caloric restriction period. Blood samples were taken before and after the caloric restriction at basal conditions and 30 min post-exercise. Although energy intake was reduced by about 33%, the contribution of carbohydrates, proteins, and lipids to total energy intake during the caloric restriction was similar to the original diet.

**Results:**

The caloric restriction reduced the daily specific micronutrient intake to values lower than 90% of recommended dietary allowances. No effects were observed in blood parameters related to iron metabolism and tissue damage, glucose levels, lipid profiles, or erythrocyte fatty acid composition. In addition, oxidative damage markers decreased after the nutritional intervention. The caloric restriction intervention significantly reduced body weight and trunk, arm, and leg weights; it also caused a decrease in fat and lean body mass, the energy expenditure rate when performing a maximal exercise stress test, and the energy cost to run one meter at various exercise intensities. Furthermore, the intervention ameliorated the onset of the anaerobic phase of exercise.

**Conclusion:**

A caloric restriction improves athletes’ performance and energy efficiency, but reduces the daily intake of micronutrients; so, when caloric restriction programs are implemented micronutrient supplementation should be considered.

**Trial registration:**

The project was registered at ClinicalTrials.gov (NCT02533479).

## Background

Reducing energy intake while maintaining nutrition, so-called caloric restriction (CR), is one of the most robust interventions for increasing lifespan in a variety of species of insects and rodents, as well as in Rhesus monkeys and for inhibiting and delaying the onset of most age-related diseases [1]. The beneficial effects of CR on the cardiovascular and cerebrovascular systems; on insulin sensitivity; on resistance to various types of stress including heat, oxidative, and metabolic stresses; on enhanced immune function; and on the regulation of body weight has been evidenced to contribute to increasing health span [2]. Energy restriction is accompanied by changes in circulating hormones, mitochondrial efficiency, and energy expenditure that serve to minimize the energy deficit, attenuate weight loss, and promote weight regain [3]. CR induces mitochondrial biogenesis and bioenergetic efficiency [4], reduces mitochondrial oxygen consumption and membrane potential, and generates less reactive oxygen species, while mitochondria are still able to maintain their critical ATP production [4]. CR is associated with improving physical fitness in rodents with respect to ad libitum-fed mice [5].

Optimal body composition provides a competitive advantage in a variety of sports. Athletes aiming to improve their strength-to-mass ratio and locomotor efficiency commonly have to reduce their weight [3]. The inclusion of an exercise component in weight loss programs for overweight and obese subjects is now standard [6]. A CR intervention could be useful for athletes looking to control their body weight and also to enhance their physical performance. It has been pointed out that in addition to weight reduction, CR can also improve a cyclist’s power-to-weight ratio without compromising endurance cycling performance [7]. Evidence of the ability of CR to enhance physical performance in athletes is scarce [7], although there are many studies on the use of CR diets for weight reduction in obese men and women [8].

CR in humans has been carried out in a variety of ways. It has been done through a reduced consumption ration and a nutrition plan adjusted to balanced-diet guidelines. Intermittent fasting (IF), that is, diets with reduced meal frequencies such as every-other-day fasting or every-other-day feeding, can have similar effects on life span and health as they provide a reduction in energy intake while maintaining nutrition [2]; it could be easier for athletes who train daily to adhere to this kind of diet rather than CR diets based solely on low daily caloric intakes. Restricting dietary energy intake could influence the total intake of essential nutrients such as vitamins, minerals, and essential amino acids, but principally essential fatty acids, rather than negatively influencing the physical performance and health of athletes.

The aim of this study was to evaluate the effects of every-other-day feeding CR interventions on the body composition and physical performance parameters (during maximal exercise tests) of well-trained athletes. We also estimate the possible impact of this CR intervention on the dietary deficiencies of micronutrients and essential fatty acids. We have previously provided evidence that a well-balanced diet supplemented with almond and olive oil-based docosahexaenoic acid and vitamin E-enriched beverages for one month does not alter athletes’ performance parameters during maximal exercise tests [9].

## Materials and methods

### Participants and study design

A simple one-centre study was performed on 12 (1 dropout) healthy males. The inclusion criteria were: age (18–50 years old), sex (male), being a non-smoker, maintaining a balanced diet, and performing physical activity > 3 but < 6 days per week. The sample size was calculated taking into account that a weight loss of 5% was significant with a 95% of confidence. The participants carried out a CR of 30–40% with respect to their usual diet, three alternate days per week for six weeks. Both before and after the every-other-day fasting CR period, the participants took a maximal exercise stress test. For each participant, one blood sample was obtained at the beginning and another at end of the every-other-day fasting intervention at basal conditions and 30 min after acute maximal exercise. All of the participants were informed of the purpose and demands of the study before providing their written consent to participate. The protocol complied with the Declaration of Helsinki for research on human subjects and was approved by the Clinical Research Ethics Committee at the Direcció General de l’Esport of the Catalonian Sports Council. The project was registered at ClinicalTrials.gov (NCT02533479).

All athletes also take part in a previous nutritional intervention study performed in order to evaluate the effects of the energy-balanced diet or dietary supplementation with functional beverages for one month training on physical performance of the athletes [9].

### CR prescription

Every participant was interviewed on their dietary, living, and training habits. Daily energy demands were calculated for each participant by taking into account the individual’s resting metabolic rate, body weight, and physical activity per week [10]. The calculated energy demands (mean ± sd) of participants were 2351 ± 156 Kcal/day distributed between a resting metabolic rate of 1862 ± 119 Kcal/day and physical activity of 3426 ± 1147 Kcal/week. Dietary habits were assessed using a 7-day dietary record which tracked all foods and fluids consumed, portion sizes, how foods were prepared, and how consumption habits were distributed throughout the day. Athletes VO_2max_ at the exhaustion was 42.6 ± 1.6 before CR intervention and 40.8 ± 1.1 after CR intervention without any significant difference From this information, a diet analysis was performed using a computer program based on CESNID food composition Tables [11].

Over a period of six weeks, the participants practiced an every-other-day fasting CR program, decreasing calorie intake by 33% with respect to their usual diets. The participants restricted their habitual diets for three alternate days each week and on the other four days participants’ dietary intakes were the same as they were at the beginning of the study without changes to caloric intake or distribution of meals.

Adherence to the nutritional intervention program was assessed using a 7-day dietary record during the last week of the intervention. All foods and fluids consumed, portion sizes, how foods were prepared, and how consumption habits were distributed throughout the day were recorded. From this information a dietary analysis was performed using a computer program based on CESNID food composition Tables [11].

### Densitometry

Densitometry was performed by Lunar IDXA (General Electric, USA) following the manufacturer’s details. Cutoffs and references were established according previously published models [12–14].

### Total body water content

Total body water content was measures by electric impedance using a two frequencies method previously described [15–17] .

### Exercise stress test energy efficiency

Each subject performed an incremental maximal test until exhaustion on a motorized treadmill (EG2, Vitoria, Spain) in order to determine his maximal oxygen consumption (VO2max) using a computerized metabolic chart (Master Screen CPX,Erich Jaeger, Wuerzburg, Germany). The protocol for the exercise test followed is previously described [9]. Each participant performed a maximal exercise test on a treadmill after fasting overnight at the beginning and again at the end of the nutritional intervention. The athletes spent three consecutive 5-min intervals running at 50%Vmax, 60%Vmax, and 70%Vmax, and then they ran at their upper anaerobic threshold running rate until reaching exhaustion. A continuous calculation of oxygen consumed (mL/min) and CO_2_ exhaled (mL/min) was made, and energy expenditure per minute (Kcal/min) at each running speed was calculated using Weir’s eq. [18]. Energy expenditure per meter run (Cal/m) was calculated using oxygen consumed (mL/min) and running rates (m/min) in Mora’s eq. [19].

### Experimental procedure

Venous blood samples were obtained in basal and 30 min after each exercise test from the antecubital vein of participants with vacutainers containing EDTA (ethylenediaminetetraacetic acid) as anticoagulant (6 mL) to obtain plasma and also to purify erythrocytes following an adaptation of the method described elsewhere [20]. Others venous blood samples were obtained to determine blood cells counts and plasma markers of nutritional status. Erythrocytes counts, hemoglobin, hematocrit, platelets counts, glucose, urea, uric acid, creatinine, bilirubin, calcium, cholesterol total, HDL, LDL, triglycerides, iron, transferrin, transferrin iron, transferrin saturation index, ferritin, vitamin D, the enzymes activities of glutamate, pyruvate transaminase, oxaloacetate transaminase, gamma-glutamyl-transferase, creatine kinase were determined by standardized clinical analytical methods.

### Lactate determination

Blood lactate was measured using a microsample of blood (20 μL) was taken from the ear while athletes were performing stress test. Blood samples were obtained at 50%, 60%, 70% of Vmax, and immediately at the end of the last bout to exhaustion (Dr. Lange®, Berlin, Germany).

### Malondialdehyde (MDA) determination

MDA levels as marker of lipopeoxidative damage were analyzed using a method previously described [21].

### Determination of erythrocyte FAs profile

Erythrocyte fatty acids composition was determined by gas chromatography/mass spectrometry using the method by Lepage and Roy [22] after total lipid extraction using the method of Folk [23] and its transformation to methyl esters by reaction with acetyl chloride. Non-esterified heptadecanoic acid (Nu-Chek Prep, Mn) in hexane was then added as an internal standard and it also containing 0.01% butylhydroxytoluene as an antioxidant. A methyl ester peaks were identified through mass spectra and by comparing the elution pattern and relative retention times of FA methyl esters. The results are expressed in relative amounts (percentage molar of total FAs).

### Statistical analysis

Statistical analysis was carried out using the Statistical Package for Social Sciences (SPSS v.21.0 for Windows). Results are expressed as mean ± standard error of the mean (SEM). The statistical significance of the data was assessed by student’s t-test for unpaired data and *p* < 0.05 was considered statistically significant.

## Results

All athletes followed an every-other-day feeding schedule in order to induce a dietary CR. The energy demands of each participant (2351 ± 156 Kcal/day) were well balanced with their energy intakes before the beginning of the intervention (2292 ± 137 Kcal/day) (Table 1). The CR habits followed by the athletes for six weeks decreased their energy intake by about 33% of their energy demand, reaching an intake of 1.537 ± 84 Kcal/day, which was significantly lower than their initial unrestricted energy intake. The reduced energy intake resulted in a reduction of daily carbohydrate, protein, and lipid intakes, each being affected differently. While, carbohydrate and lipid intakes were significantly reduced, by 33% and 35% respectively, protein intake was reduced by only about 25% with respect to the initial unrestricted diet levels. In spite of the different levels of reduction in carbohydrate, protein, and lipid intakes, their contribution to total energy intake during the CR intervention was similar to their contribution in the original unrestricted diet. The contribution of animal and vegetable proteins to total protein intake during the CR intervention was maintained at the original unrestricted diet levels; similarly, the contribution of SFA and PUFA to total lipid intake during the CR intervention was maintained, but the MUFA component was significantly higher than it was in the original unrestricted diet. Cholesterol intake was reduced by about 35% during the intervention.

**Table 1:** Energy and macronutrient intake during calorie restriction intervention

	Pre-RC	Post-RC
Energy *(Kcal)*	2292 ± 137	1537 ± 84 *
Carbohydrate (CHO) *(g/day)*	230 ± 16	155 ± 2.0 *
Polysaccharide *(g/day)*	121 ± 10	80.6 ± 7.3*
Simple CHO *(g/day)*	82.7 ± 9.0	62.2 ± 4.9*
CHO/Bogy weight *(g/Kg)*	2.8 ± 0.2	2.0 ± 0.15*
Protein *(g/day)*	103 ± 5.0	77.0 ± 3.7 *
Animal *(% Protein)*	70.8 ± 3.8	71.5 ± 4.7
Vegetal *(% Protein)*	29.2 ± 7.9	28.5 ± 6.1
Protein/Body weight *(g/Kg)*	1.2 ± 0.1	1.0 ± 0.05*
Lipids *(g/day)*	97.3 ± 6.6	63.3 ± 5.0 *
SFA *(% Lipids)*	37.7 ± 1.2	35.3 ± 1.3
MUFA *(% Lipids)*	44.7 ± 1.8	48.9 ± 1.1 *
PUFA *(% Lipids)*	17.5 ± 2.9	15.8 ± 5.1
Cholesterol *(mg/day)*	374 ± 2.4	245 ± 1.7 *

Pre-RC and Post-RC: previous and at the end of calorie restriction intervention. Statistical analysis: Student’s test for unpaired data. (*) Significant differences between placebo and experimental, *p* < 0.05

CR also reduced athletes’ daily micronutrient intake (Table 2), leading to values lower than recommended dietary allowances (RDA) for athletes (CESNID) [11]. The daily intake of magnesium, potassium, zinc, folic acid, and calciferol were about 60–77% lower than RDAs for athletes in the original unrestricted diet, and these values were reduced to 48–67% of RDAs with the CR. Additionally, the intervention significantly reduced daily intake of micronutrients and led to values lower than 90% of RDAs for iron, niacin, riboflavin, pyridoxine, and vitamins A and D. The CR diet created a situation of low micronutrient and vitamin intake, which, if maintained over the long term, could compromise athletic performance. However, the possible micronutrient intake deficiency induced by the one-month 33% CR did not compromise physical performance; on the contrary it ameliorated several parameters related to physical performance. We cannot plan a CR diet that would lead to a micronutrient deficiency without providing specific dietary supplementation that would rectify micronutrient intake.

**Table 2:** Micronutrient intake during calorie restriction intervention

	Pre-RC	RDA (%)	Post-RC	RDA (%)
Fiber (g)	16.4 ± 1.4		13.6 ± 0.8 *	
Calcium (mg)	848 ± 81	106%	729 ± 68	91%
Iron (mg)	12.2 ± 1.3	122%	8.5 ± 1.5 *	86%
Magnesium (mg)	270 ± 16	77%	223 ± 8 *	64%
Sodium (mg)	2616 ± 208		1922 ± 169 *	
Potassium (mg)	2599 ± 168	74%	2014 ± 91 *	58%
Phosphorus (mg)	1135 ± 115	162%	1056 ± 101	151%
Zinc (mg)	9.00 ± 0.65	60%	7.10 ± 0.33 *	47%
Carotenoids (μg)	1978 ± 312		1985 ± 226	
Folic acid (μg)	254 ± 37	64%	217 ± 21	54%
Niacin (mg)	22.3 ± 2.1	112%	17.0 ± 1.0 *	85%
Retinoid (μg)	613 ± 241		150 ± 17	
Riboflavin (mg)	1.60 ± 0.18	92%	1.30 ± 0.28	75%
Thiamine (mg)	1.31 ± 0.13	113%	1.12 ± 0.08	97%
Retinol (μg)	919 ± 260	92%	482 ± 42	48%
Pyridoxine (mg)	2.53 ± 0.65	139%	1.45 ± 0.08	83%
Calciferol (μg)	2.96 ± 0.50	77%	1.62 ± 0.25 *	58%
Tocopherol (μg)	9.22 ± 0.68	311%	7.00 ± 0.73 *	176%
Cobalamin (μg)	6.20 ± 1.20	152%	3.51 ± 0.25 *	125%
Ascorbic acid (mg)	91.1 ± 13.5	106%	74.7 ± 10.0	91%

RDA recommended daily allowance, CESNAD 2010. Statistical analysis: Student’s test for unpaired data. (*) Significant differences between placebo and experimental, *p* < 0.05

The CR intervention significantly reduced body weight (4.40%) as well as trunk, arm, and leg weights (Table 3). The percentage of weight reduction in the arms (3.70%) was lower than the percentage of weight reduction in the legs (4.91%), which in turn was lower than the percentage of weight reduction in the trunk (6.4%). Body fat mass was reduced by 15.1% after the six weeks of CR, and it was the main cause of body weight loss; lean body mass was only reduced by 2.91%. The main site of fat mass loss was in the trunk (17.4%); the legs lost fat mass (10.4%) in a similar proportion to the arms (9.07%). The legs were the main area of lean body mass loss (3.47%), whereas the trunk and arms lost similar percentages of lean mass (2.45% and 2.62%, respectively). The bone mineral content in the body, legs, and arms, maintained initial values, but it significantly decreased (0.014%) in trunk. The measurement of total body water (TBW) significantly decreased (*P* ≤ 0.001) from 45.4 ± 2.6 kg to 42.9 ± 3.6 kg after the CR intervention. CR also significantly changed intracellular and extracellular water contents. Extracellular content increased significantly (*P* = 0.038) from 19.2 ± 1.8 kg to 20.1 ± 1.6 kg. Intracellular water content significantly decreased (*P* = 0.003) from 26.2 ± 72.7 kg to 22.8 ± 3.9 kg. The body weight losses induced by the 33% CR in well-trained athletes were mainly due to TBW losses, although extracellular water increased and intracellular water decreased their body content after the CR.

**Table 3:** Effects of calorie restriction on body composition

Body	Pre-CR	Post-RC
Total weight (kg)	81.0 ± 1.9	77.4 ± 1.9 *
Tissue weight (kg)	78.9 ± 1.9	74.1 ± 1.9 *
Fatty body mass (kg)	20.5 ± 1.4	17.4 ± 1.3 *
Lean body mass (kg)	58.4 ± 1.2	56.7 ± 1.3 *
BMC (kg)	2.82 ± 0.07	2.81 ± 0.07
Arm		
Fat (g)	1797 ± 115	1634 ± 116 *
Lean (g)	7148 ± 219	6961 ± 216 *
BMC (g)	466 ± 11	468 ± 12
Total mass (g)	9412 ± 257	9064 ± 267 *
Legs		
Fat (g)	6590 ± 481	5904 ± 425 *
Lean (g)	20,537 ± 477	19,825 ± 537 *
BMC (g)	1313 ± 34	1315 ± 34
Total mass (g)	28,442 ± 752	27,045 ± 737 *
Trunk		
Fat (g)	10,330 ± 829	8532 ± 748 *
Lean (g)	27,129 ± 546	26,463 ± 546 *
BMC (g)	984 ± 32	970 ± 32 *
Total mass (g)	38,444 ± 1060	35,965 ± 947 *

Statistical analysis: Student’s test for unpaired data. (*) Significant differences between placebo and experimental, *p* < 0.05

No significant changes attributable to the CR were observed in parameters related to iron metabolism, such as erythrocyte counts, hematocrit, blood hemoglobin, plasma iron, ferritin, bilirubin, transferrin saturation, or transferrin iron, although the percentage of transferrin slightly decreased (by about 3.3%) after the CR (Table 4). The six weeks of CR did not cause tissue damage, considering that serum activities of GPT, GOT, GGT, and Creatin kinase were maintained. The CR did not influence plasma glucose levels or nitrogen metabolism markers, such as plasmatic urea, creatinine, or urate levels. Lipid metabolism was influenced by the CR. Circulating levels of cholesterol and triglycerides were significantly lower after the six-week period compared to previous levels, but HDLs and LDLs maintained their pre-CR values. Calcium remained level, but vitamin D increased in plasmatic values after the six weeks of CR.

**Table 4:** Calorie restriction effects on blood cells and plasmatic markers of nutritional status

	Pre-CR	Post-CR	*P* values
Erythrocytes (10^6^ cells/mL)	5.12 ± 0.10	5.14 ± 0.12	0.910
Hemoglobin (g/dL)	15.1 ± 0.4	15.2 ± 0.5	0.791
Hematocrit (%)	44.9 ± 0.9	45.1 ± 1.2	0.847
Iron (μmol/L)	87.9 ± 12	104 ± 17	0.416
Transferrin (g/L)	266 ± 10	257 ± 9 *	0.032
Transferrin iron (μmol/L)	66.6 ± 2.5	64.5 ± 2.3	0.138
Transferrin saturation (%)	24.0 ± 3.4	28.8 ± 4.9	0.396
Ferritin (ng/mL)	158 ± 19	236 ± 64	0.435
Glucose (mg/dL)	84.1 ± 1.2	80.9 ± 2.4	0.250
Urea (mg/dL)	31.2 ± 4.3	35.5 ± 2.3	0.194
Uric acid (mg/dL)	5.82 ± 0.42	5.93 ± 0.33	0.672
Creatinine (mg/dL)	1.06 ± 0.06	1.04 ± 0.08	0.292
Bilirubin (mg/dL)	0.92 ± 0.17	0.94 ± 0.17	0.882
GPT (U/L)	23.6 ± 1.8	25.1 ± 3.4	0.588
GOT (U/L)	21.8 ± 2.4	20.4 ± 2.1	0.467
GGT (U/L)	24.3 ± 5.5	33.9 ± 12.4	0.253
Creatinequinase (U/L)	178 ± 33	165 ± 42	0.568
Cholesterol total (mg/dL)	184 ± 9	176 ± 10 *	0.049
HDL (mg/dL)	52.9 ± 3.1	53.2 ± 2.4	0.807
LDL (mg/dL)	110 ± 8	107 ± 10	0.488
Triglycerides (mg/dL)	99.4 ± 9.3	85.4 ± 10 *	0.043
Vitamin D (ng/mL)	21.6 ± 4.9	25.3 ± 4.9 *	0.001
MDA (μmol/10^6^ cells)	49.8 ± 1.8	42.0 ± 1.2 *	0.026

Statistical analysis: Student’s test for unpaired data. (*) Significant differences between placebo and experimental, *p* < 0.05

Erythrocyte fatty acid composition (Table 5) was mostly maintained, as similar levels were seen before and after the intervention; although C20:5 n3 significantly increased by 43%, and C22:0 and C24:0 significantly decreased (by 15% and 12.6%, respectively) in erythrocytes.

**Table 5:** Erythrocyte fatty acid composition

	Pre-CR	Post-CR
C14:0 (%)	0.289 ± 0.021	0.270 ± 0.025
C:16 (%)	25.5 ± 0.7	25.9 ± 1.2
C16:1n7 (%)	0.264 ± 0.025	0.240 ± 0.032
C:18 (%)	18.9 ± 0.4	18.9 ± 0.6
C18:1n9 (%)	13.8 ± 0.4	13.9 ± 0.3
C18:1n7 (%)	1.07 ± 0.04	1.16 ± 0.07
C18:2n6 (%)	8.82 ± 0.38	8.36 ± 0.33
C18:3n6 (%)	0.116 ± 0.041	0.139 ± 0.021
C18:3n3 (%)	0.0763 ± 0.0217	0.0712 ± 0.0136
C18:4n3 (%)	0.0285 ± 0.0129	0.0179 ± 0.0119
C20:0 (%)	0.517 ± 0.021	0.475 ± 0.018
C20:1n9 (%)	0.265 ± 0.014	0.285 ± 0.012
C20:2n6 (%)	0.243 ± 0.012	0.247 ± 0.012
C20:3n6 (%)	1.12 ± 0.11	1.02 ± 0.10
C20:4n6 (%)	10.7 ± 0.8	11.3 ± 1.2
C20:5n3 (%)	0.588 ± 0.052	0.845 ± 0.165*
C22:0 (%)	1.64 ± 0.08	1.39 ± 0.08*
C22:1n9 (%)	0.288 ± 0.133	0.394 ± 0.193
C22:4n6 (%)	2.04 ± 0.21	2.03 ± 0.24
C22:5n6 (%)	1.23 ± 0.12	1.34 ± 0.18
C24:0 (%)	4.45 ± 0.22	3.89 ± 0.18*
C22:6n3 (%)	2.90 ± 0.27	3.14 ± 0.38
C24:1n9 (%)	4.70 ± 0.17	4.34 ± 0.29
SFA (%)	51.3 ± 1.3	50.8 ± 1.9
MUFA (%)	20.4 ± 0.6	20.3 ± 0.6
PUFA (%)	28.2 ± 1.7	28.9 ± 2.3
n-6 (%)	23.4 ± 1.3	23.5 ± 1.7
n-3 (%)	4.82 ± 0.40	5.42 ± 0.60
n-6/n-3	5.13 ± 0.32	5.26 ± 0.87
n-3 INDEX	3.49 ± 0.31	3.99 ± 0.52

Statistical analysis: Two-way ANOVA. *p* < 0.05 (CR) Significant effect of calorie restriction One-Way ANOVA. *p* < 0.05. (*) Significant differences between pre- and post-calorie restriction

**Fig. 1:**
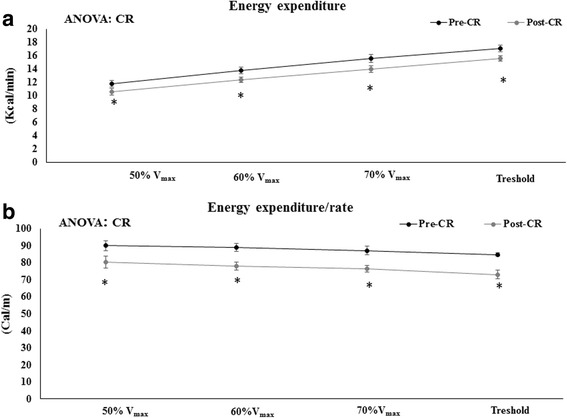
Effects of Caloric Restriction on energy expenditure and on energy expenditure rate. **a** Represents energy expenditure. **b** Represents energy expenditure per meter. Significant differences between Pre-CR and Post-CR (Caloric Restriction) *P* < 0.05 unpaired‘t’ student test

**Fig. 2:**
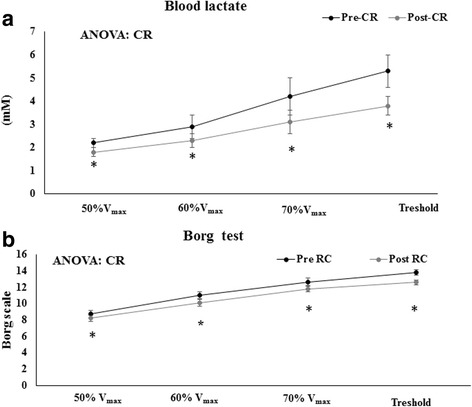
Effects of caloric restriction on lactate blood levels and on Borg test. **a** Represents lactate blood concentration. **b** Represents Borg test results. *Significant differences between Pre-CR and Post-CR (Caloric Restriction) *P* < 0.05 unpaired‘t’ student test

The CR reduced athletes’ heart rates when performing the same exercise at the same duration and level of power (Table 6). Similarly, the energy expenditure required for running at 50%Vmax, 60%Vmax, and 70%Vmax and for running at threshold until exhaustion was about 10% lower after the CR intervention than before it (Fig. 1a). This more efficient use of energy when carrying out physical activity associated with the CR was also evident when the energy cost to run a unit of distance was calculated: the energy expenditure required to run one meter at 50%Vmax, 60%Vmax, 70%Vmax, and the threshold maximal running power was about 10.7–13.8% lower after the six weeks of CR when compared to previously observed values related to an unrestricted diet (Fig. 1a and b). In addition, the blood lactate levels during each running period were significantly lower after six weeks of CR when compared to pre-CR levels. The CR ameliorated the onset of the anaerobic phase of exercise with respect pre-intervention values. In this sense, the perception of exertion, as indicated by the Borg index, also decreased after the CR when compared to values observed previously, under an unrestricted diet (Fig. 2a and b).

**Table 6:** Effects of Calorie restriction on markers of physical performance

		50%V_mx_	60% V_max_	70%V_max_	Threshold
Running Rate *(Km/h)*	Pre-CR	7.70 ± 0.3	9.10 ± 0.3	10.4 ± 0.3	11.7 ± 0.3
	Post-CR	7.70 ± 0.3	9.10 ± 0.3	10.4 ± 0.3	11.7 ± 0.3
Heart Rate *(Beats/min)*	Pre-CR	133 ± 5	149 ± 4	160 ± 4	171 ± 4
	Post-CR	127 ± 6	142 ± 5*	153 ± 5 *	161 ± 3*

*Significant differences between Pre-CR and Post-CR (Caloric Restriction) *P* < 0.05 unpaired ‘t’ student test

## Discussion

The main feature of the present study is that after six weeks of participating in a CR program based on intermittent partial fasting, athletes’ physical performance was enhanced. Previous results obtained in a similar study provide evidence of the null effects of one month of training on performance parameters determined during a maximal exercise test on athletes that consumed well-balanced diets or even supplemented their diets with a functional beverage [9]. In addition, the CR via every-other-day fasting allowed for body weight to be controlled, as mainly body fat was lost, although some lean mass loss was also observed. The CR decreased lipid, carbohydrate, and protein intake by 35%, 33%, and 25%, respectively. The six-week CR resulted in slight lean body mass loss, similar to what was found in other CR trials with a 40% calorie reduction and protein intake around 1 g/kg of body weight for two weeks [24]. The loss of lean body mass during weight reduction is considered a negative effect that could compromise performance [25]. Lean body mass loss during the CR via intermittent partial fasting could probably be avoided by increasing protein intake to around 2.3 g/kg of body weight [24] or by supplementing the diet with branched-chain amino acids that maintain lean mass while promoting the loss of fat mass [26]. Regardless of how they do it, athletes must aim to preserve lean body mass during weight reduction [24, 25]. To increase dietary protein intake while in a negative energy balance would come at the “expense” of another macronutrient, such as fat.

The CR led to a micronutrient and vitamin intake below RDAs for athletes, which could have compromised their exercise performance. In this sense, CR intervention programs might consider supplementing diets with vitamins and micronutrients, such as iron, magnesium, potassium, zinc, folate, riboflavin, pyridoxine, and vitamins A and C. However, blood markers of nutritional status in athletes, such as those related to iron metabolism, calcium and vitamin D, glucose, markers of nitrogen handling, or those related to tissue damage, maintained the same levels before and after the intervention. This result has been observed in other trials with hypocaloric diets [24].

The low fat intake associated with the CR could alter the availability of omega-3 and omega-6 polyunsaturated fatty acids and lead to not meeting daily requirements. The fatty acid composition of erythrocyte is a good marker for assessing the efficacy of nutritional intervention trials in incorporating dietary fatty acids [21]. The presence of different fatty acids in the diet and lifestyle factors, such as exercise and obesity, influence the incorporation of the acids into different tissues and erythrocyte membranes [21]; the erythrocyte content of the omega-3 and omega-6 essential fatty acids were maintained or even increased after the six weeks of CR. The possible lack of fatty acid availability during six-week CR did not affect fatty acid content in erythrocyte membranes. The 35% reduction in lipid intake seen under the CR program caused lower triglyceride and cholesterol plasma levels than were observed with the unrestricted diet, but erythrocyte maintained its omega-3 and omega-6 polyunsaturated fatty acids content. Additionally, the CR lessened oxidative damage in plasma lipids. It has been pointed out that a CR decreases mitochondrial electron flow as well as proton leaks in mammalian cells, and attenuates muscle damage caused by intracellular reactive oxygen species [4, 27]. We actually provide evidence that a CR reduces oxidative damage in circulating lipids and blood vessels along with reducing circulating triglyceride and cholesterol levels.

The CR intervention significantly reduced body, trunk, arm, and leg weights; it mainly reduced body fat mass, but a small yet significant reduction in lean body mass was also observed. Reducing body weight is a goal for many athletes [25]. Either rapid or gradual body weight reduction techniques have been used to control body weight in athletes with varied results on physical performance. Aerobic endurance capacity decreases after rapid body weight reduction. A severe CR of 67% to 90% of energy demands and dehydration during 48 h reduces exercise capacity in the heat compared to an adequate energy control trial [28]. It also causes detrimental effects in a 30-min treadmill time-trial session in temperate conditions [29]; affects health, muscle performance, and energy; and alters perceived exertion and dynamic postural control [30]. Severe CR also results in a large reduction in body mass that appears to be mostly explained by a rapid reduction in body water stores [31]. Gradual body weight reduction via a smaller CR induces more consistent body weight losses over a CR period of more than one week. In our study, a CR of 33% via intermittent partial fasting over six weeks led to about 0.9 kg/week of weight loss. This finding is in line with others who have observed a body weight loss of 1 kg/week with an energy intake reduction of about 25 Kcal/kg/day [25], and the losses are likely reflected in a loss of body fat and muscle [31]. We also observed a decrease in TBW content, stemming mainly from the intracellular water compartment, which represented 69% of total body weight loss. The CR also altered the water distribution between the body’s intracellular and extracellular compartments: the intercellular compartments lost water while extracellular water content increased after the intervention.

Sedentary, overweight men and women, at 55 years of age, with confirmed metabolic syndrome saw body weight losses after a 12-week 30% CR. A co-intervention with moderately intense aerobic exercise enhanced the weight losses induced by the 30% CR; the exercise led to a greater reduction in central adiposity and trunk fat mass, and it improved maximal oxygen consumption [32]. Short-term hypoenergetic weight loss programs could maintain lean body mass in young healthy athletes who have a protein intake around 2.3 g/day/kg of body weight [24].

It has been demonstrated that VO2max is increased after a body weight reduction [25]; in this sense, we have detected an improvement in physical performance markers, such as heart rate, lactate levels, fatigue perception (Borg index), and the energy expenditure required to run a meter, that are not dependent exclusively on changes in total body mass [33]. Previous studies with well-balanced diets or even after one month of consuming functional beverage supplements provided no evidence suggesting that these nutritional habits have an effect on physical performance parameters such as lactate levels [9]. Additionally, we observed a decrease in lean body mass as a consequence of the CR; however, this decrease did not involve any impairment of physical performance parameters. This fact could indicate that athletes should have an ideal lean body mass in order to produce maximal physical performance. To sum up, higher muscle mass does not necessarily indicate better physical performance. Furthermore, we detected several changes in energy expenditure and in energy efficiency as a consequence of the CR; this fact could be explained by considering that CR might cause increased mitochondria efficiency [4].

This study was limited by the reduced sample size of the population studied and the participants’ characteristics as Olympic athletes, as well as the duration of the study (weeks).

So, we observed that a 33% CR via every-other-day fasting significantly reduced daily micronutrient intake to 90% of RDA values; it also reduced body weight by 4.4%, fat body mass content by 15.1%, and lean body mass by 2.91%. Moreover, the CR reduced plasma triglycerides by 14.1% and cholesterol by 4.3% with respect to control values, and it also reduced MDA plasma levels by 15.7%. Physical performance parameters, such as heart rate, lactate levels, fatigue perception (Borg index), were significantly improved as a consequence of the CR, which ameliorated the onset of the anaerobic phase of exercise. Moreover, the CR decreased the energy expenditure required to run one meter and improved energy efficiency. However, when implementing a CR, a micronutrient supplement should also be considered.

## Abbreviations

CESNID: Centro Enseñanza Superior de Nutricion y Dietetica
CR: Caloric restriction
EDTA: Ethylenediaminetetraacetic Acid
FAs: Fatty acids
HDL: High density lipoproteins
IF: Intermittent Fasting
LDL: Low density lipoprotein
MDA: Malondialdehyde)
MUFA: Monounsaturated Fatty Acids
PUFA: Polyunsaturated Fatty Acids
RDA: Recommended Dietary Allowances
SFA: Saturated Fatty Acids
VO2max: Maximal Oxygen Consumption
